# Ambient-task combined lighting to regulate autonomic and psychomotor arousal levels without compromising subjective comfort to lighting

**DOI:** 10.1186/s40101-021-00258-w

**Published:** 2021-08-09

**Authors:** Junichiro Hayano, Norihiro Ueda, Masaya Kisohara, Yutaka Yoshida, Emi Yuda

**Affiliations:** 1 Department of Medical Education, Nagoya City University Graduate School of Medical Sciences, 1 Kawasumi Mizuho-cho Mizuho-ku, Nagoya, 467-8601 Japan; 2 Nagoya City University Graduate School of Design and Architecture, Nagoya, 464-0083 Japan; 3 Center for Data-driven Science and Artificial Intelligence, Tohoku University, 41 Kawauchi, Aoba-ku, Sendai, 980-8576 Japan

**Keywords:** autonomic nervous system, blue light, combined lighting, color temperature, continuous performance, heart rate variability, intrinsically photosensitive retinal ganglion cell, non-image forming function, light emitting diode, psychomotor vigilance, subjective comfort

## Abstract

**Background:**

Although evidence of both beneficial and adverse biological effects of lighting has accumulated, biologically favorable lighting often does not match subjectively comfortable lighting. By controlling the correlated color temperature (CCT) of ambient lights, we investigated the feasibility of combined lighting that meets both biological requirements and subjective comfort.

**Methods:**

Two types of combined lightings were compared; one consisted of a high-CCT (12000 K) light-emitting diode (LED) panel as the ambient light and a low-CCT (5000 K) LED stand light as the task light (high-low combined lighting), and the other consisted of a low-CCT (4500 K) LED panel as the ambient light and the same low-CCT (5000 K) stand light as the task light (low-low combined lighting) as control. Ten healthy subjects (5 young and 5 elderly) were exposed to the two types of lighting on separate days. Autonomic function by heart rate variability, psychomotor performances, and subjective comfort were compared.

**Results:**

Both at sitting rest and during psychomotor workload, heart rate was higher and the parasympathetic index of heart rate variability was lower under the high-low combined lighting than the low-low combined lighting in both young and elderly subject groups. Increased psychomotor alertness in the elderly and improved sustainability of concentration work performance in both age groups were also observed under the high-low combined lighting. However, no significant difference was observed in the visual-analog-scale assessment of subjective comfort between the two types of lightings.

**Conclusions:**

High-CCT ambient lighting, even when used in combination with low-CCT task lighting, could increase autonomic and psychomotor arousal levels without compromising subjective comfort. This finding suggests the feasibility of independent control of ambient and task lighting as a way to achieve both biological function regulation and subjective comfort.

## Background

The functions of food are divided into the primary function of "nutrition" and the secondary function of "preference" and deliciousness, as well as the "bioregulatory function" as a tertiary function [1]. Similarly, the functions of light include bioregulatory function [2–11] in addition to function as light for work at night or in indoor environments, function as signs, and function for the artistry and rendition of space. Among the bioregulatory function of light, the non-image forming effects of blue wavelength light through the intrinsically photosensitive retinal ganglion cells (ipRGC) [12, 13] are attracting attention [8, 14–17]. The non-image forming effects of blue light include important biological regulations, such as increased vigilance [18, 19], autonomic nervous arousal [20, 21], and circadian clock adaptation to the environment [22–25], but that is why excessive exposure to blue light, especially before bedtime, could adversely affect sleep due to improper arousal and circadian rhythm disturbances [26–30]. To utilize the bioregulatory function of light appropriately, it is necessary to optimize the illuminance, correlated color temperature (CCT), and the amount of melanopsin-stimulating photo-spectral component according to the activity schedules and time zone. However, biologically required lighting characteristics often do not match subjectively comfortable lightings or those required for the activities of individual people.

In this study, we examined the possibility of combined lighting consisting of high-CCT ambient lighting and low-CCT task lighting as a method to meet the biological requirements and subjective comfort. The effects of the combined lighting on objective arousal level, psychomotor performance, and subjective comfort were compared with those when used low-CCT lights for both ambient and task lighting. The ambient lighting was designed to illuminate from a direction perpendicular to the subject's visual axis. In a previous study, we examined whether the autonomic responses to blue light depends on the angle of incidence to the eye in healthy subjects, but no significant difference was observed in autonomic indices of heart rate variability for a wide range of angles of incidence from -90° to +90° [31]. This suggests that the biological effects of the non-imaging function of blue light may be regulated separately from the subjective comfort at least that is caused by the image-forming function by choosing different directions of light into the eye.

## Methods

### Subjects

The subjects were recruited with the following inclusion criteria: healthy men or women who (1) were between 20 and 30 years old for young group and between 65 and 80 years old for elderly group, (2) had never been diagnosed with color vision abnormalities, (3) were not taking any medications for >two weeks, and (4) displayed a normal sinus rhythm on electrocardiogram (ECG) at rest. There were five applicants for each of the young and elderly groups (mean age ± SD, 22 ± 1 year, four males and 72 ± 2 year, five males, respectively) who met the inclusion criteria, and all applicants participated in the experiment.

### Lighting devices

**Fig. 1:**
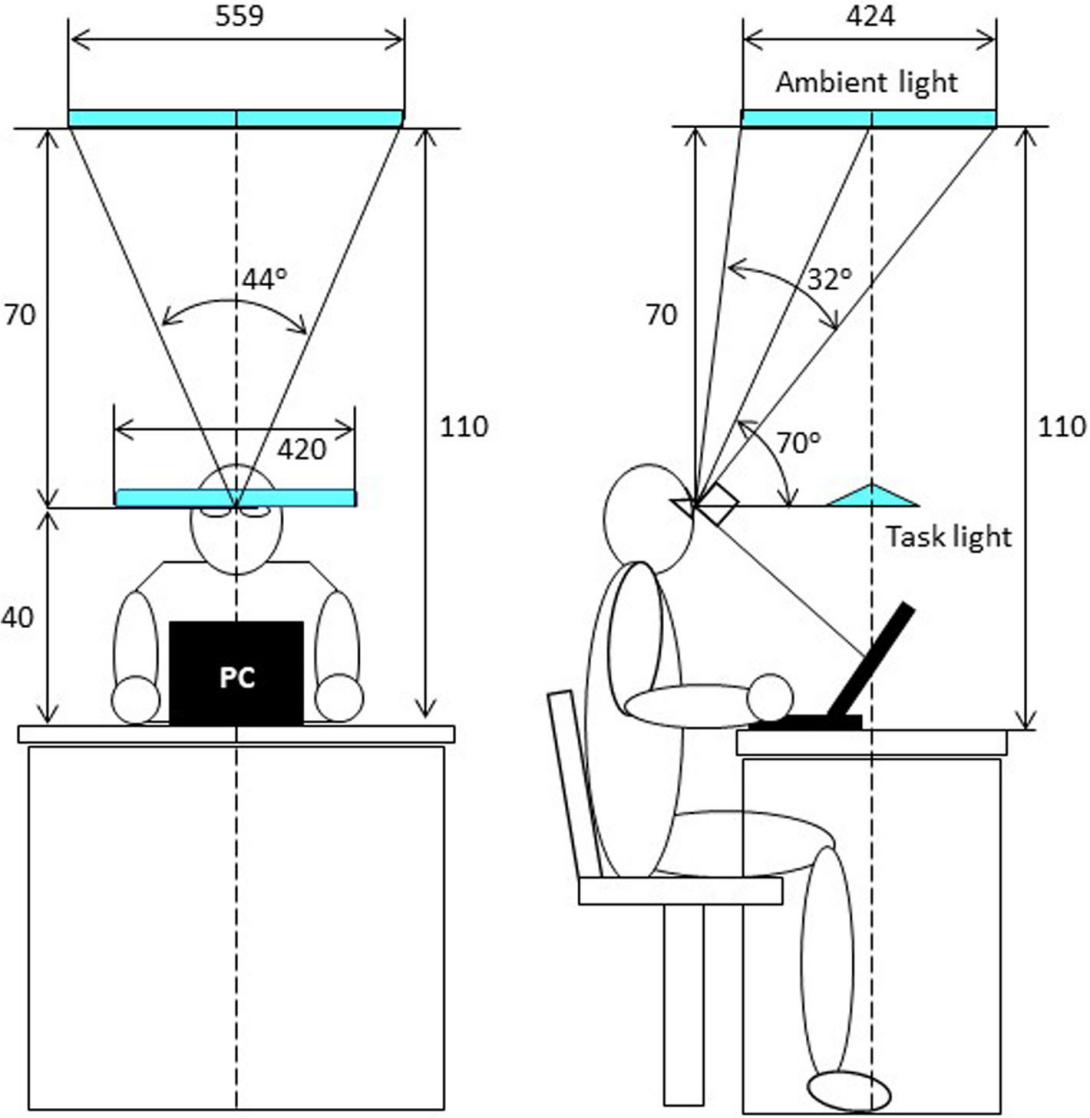
Scheme of experimental combined lighting system. The system is composed of an RGB variable light-emitting diode (LED) lighting panel for the ambient lighting and a dimmable LED stand light for the task lighting. The unit of length is mm. PC = personal computer

We compared the combined lighting of blue-enriched high-CCT ambient lighting and low-CCT task lighting (high-low combined lighting) and the combined lighting with low-CCT lights for both ambient and task lighting (low-low combined lighting). Figure 1 shows the scheme of combined lighting system used in the present study. The lighting system consisted of an ambient light panel and a task light stand. An RGB variable light-emitting diode (LED) panel (Dimming and wide toning panel light, Model SXPS0001, ENDO Lighting Corporation, Osaka, Japan) with a luminous area of 559 × 424 mm was used for the former, and a stand light (Dimming and toning task light, Model Z-10NB, Yamada Shomei Co., Ltd., Japan) was used for the latter. The ambient lighting panel was placed above the head of subjects sitting in front of a work desk. The position of the panel was adjusted so that when the subject looked at the computer display on the desk, the ambient lighting would illuminate from a direction perpendicular to the subject’s visual axis.

**Fig. 2:**
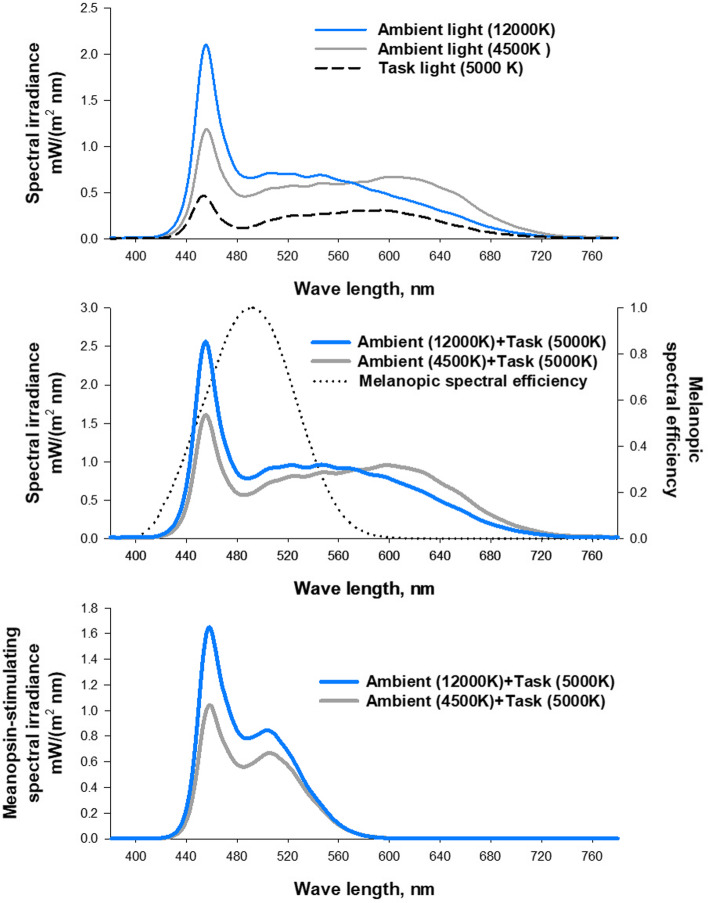
Spectral irradiances of the ambient and task lighting (top), ambient and task combined lightings (middle), and their melanopsin-stimulating fractions (bottom). The melanopsin-stimulating spectral irradiance was calculated from the melanoptic spectral efficiency curve (dotted line in the middle panel) adjusted for the effect of human pre-receptoral filtering [15, 32, 33]

Figure 2 shows the photo-spectra of the ambient and task lighting and Table 1 shows the properties of lightings measured as vertical illuminances on the desk top with a photo-spectrometer (Asensetek Lighting Passport, Model No. ALP-01, KLV Co., Ltd., Tokyo, Japan). The melanopsin-stimulating spectral irradiance calculated from the melanoptic spectral efficiency curve adjusted for the effect of human pre-receptoral filtering [15, 32, 33] were 93 and 68 mW/m^2^nm and the relative content ratios were 42% and 32% for high-low and low-low combination lighting, respectively.

**Table 1:** Characteristics of ambient, task, and combined lightings

	CCT, K	Illuminance, lx*	Irradiance mW/(m^2^nm)	Melanopsin-stimulating spectral component	
				Irradiance mW/(m2nm)	Relative content, %
Low-CCT ambient	4500	500	152	51	34
High-CCT ambient	12000	500	163	75	46
Task	5000	250	60	19	31
Low-low combined	4900	750	211	68	32
High-low combined	8250	750	223	93	42

*Desk-top vertical illuminance.

CCK = correlated color temperature.

## Study protocols

Subjects were instructed not to consume food or beverages containing caffeine or alcohol after 21:00 the previous night and to take >7 hours of sleep. All studies were performed between 13:00 and 16:15 in a calm, light-shielded, and air-conditioned (24 ± 2°C) laboratory more than 2 hours after a light meal.

For each subjects the same experiment was performed twice under the low-low combined lighting and under the high-low combined lighting on different days apart for a week. The order of two different combined lighting exposures was counter balanced among subjects. On each day, ambulatory ECG was recorded continuously during the experiment with a Holter ECG recorder (Cardy 303 pico plus, Suzuken Co., Ltd., Nagoya, Japan). Data were collected with subjects sitting in front of the work desk for five min at rest, during three kinds of psychomotor performance tests performed at an interval of one-min rest (total 28 min), and for five min at rest (Table 2).

**Table 2:** Protocols

Time	Under exposure to each combined lighting
0 - 5 min	Sitting rest
6 - 11 min	Psychomotor vigilance test (PVT)
12 - 18 min	Pedal-selective psychomotor vigilance test (PS-PVT)
19 -34 min	Visual continuous performance test (VCPT)
35 - 40 min	Sitting rest
41 - 45 min	VAS assessment of subjective feelings

### Assessment of autonomic functions

We analyzed heart rate variability to assess the effects of lighting on autonomic nervous functions. Recorded ECG were digitized continuously at 125 Hz into 10-bit data. The digitized data were analyzed off-line with analyzer software (Cady Analyzer 5, Suzuken Co., Ltd., Nagoya, Japan), by which the temporal positions of all QRS waves were detected and the annotation of QRS waves were given. After all errors in the QRS wave detection were edited, time series of the R-R interval were obtained. The R-R interval time series thus obtained were used for continuous heart rate variability analysis by complex demodulation [34, 35]. Briefly, R-R interval time series were interpolated with a horizontal-step function only using interval data consisting of consecutive QRS waves in sinus rhythm (normal-to-normal intervals) and resampled at a frequency of 2 Hz. Then, the amplitudes in the low frequency (LF, 0.04-0.15 Hz) and high frequency (0.15-0.40 Hz) bands were demodulated and averaged over every 60-sec non-overlapping window. The LF-to-HF power ratio (LF/HF) was calculated every 60-sec non-overlapping window as the squared ratio of their amplitude. The HF amplitude was used as an index of cardiac parasympathetic function and the LF amplitude was interpreted as an index affected by both sympathetic and cardiac parasympathetic function [36].

### Psychomotor performance tests

#### PVT

We employed PVT to assess the effects of lighting on sustained attention. We used validated software called PC-PVT [37] that was downloaded from http://bhsai.org/downloads/pc-pvt/ and installed in a laptop personal computer (Let’s note CF-N10, Panasonic Co., Osaka, Japan) with a computer mouse (MA-BL10BK, Sanwa Supply, Okayama City, Japan). PVT quantifies reaction time (RT) to visual stimuli. The software presented a time counter in red on a black background on the computer display. The counter appeared repeatedly, started from 0 every time, and incremented by one every millisecond until the subject clicked the computer mouse, although the counter was only displayed at intervals according to the refresh rate of the computer display. When the mouse was clicked, the last counter corresponding to RT was displayed for 1 sec and the next time counter appeared after a randomized interval between 2 and 10 sec. In this study, the test was performed with setting the parameters of anticipation at 100 ms, deadline at 65000 ms, minor lapse at 500 ms, and major laps at 1000 ms. The total trial time was set at 300 sec, during which 46 to 51 stimuli were presented.

The data were processed offline. The RT was averaged and minor and major lapse frequencies were calculated as the percentage in the total number of stimuli over the PVT session.

#### Pedal-selective psychomotor vigilance test (PS-PVT)

We used PS-PVT to evaluate the effects of lighting on psychomotor performance. We used custom made software for PS-PVT. The details of PS-PVT have been reported previously [38]. Briefly, the system consisted of a laptop personal computer (Let’s note CF-N10, Panasonic Co., Osaka, Japan) and a three-pedal foot mouse (RI-FP3BK, Route-R co., Ltd., Tokyo, Japan). Initially, two open circles with a diameter of five cm and a center-to-center distance of 15 cm were displayed horizontally on a black background on the computer display. In random order and interval, either one of the circles was lit, blue for the left circle and red for the right circle. Subjects were instructed to press a foot pedal with their right leg as prompt as possible when the circle illuminated. In parallel mode, they were asked to press the pedal ipsilateral to the lit circle, while in cross mode, they were asked to press the contralateral pedal. When the correct pedal was pressed, the light turns off immediately, but when the wrong pedal was pressed, the light did not turn off and instead buzzer sounded until the correct pedal was pressed. The trial lasted six min, during which time the mode was switched between parallel and cross every two min. During the cross mode, a cross mark appears on the display. For the PS-PVT data, averaged RT, the frequencies of false pedaling and lapse (>500 ms), and averaged correction time were calculated.

#### Visual Continuous Performance Test (VCPT)

We selected VCPT to evaluate the effects of lighting on the sustainability of work that requires concentration (response speed and attention) and its time-dependent changes. We used custom made software for VCPT. The test used a laptop personal computer (Let’s note CF-N10, Panasonic Co., Osaka, Japan) and a mouse. The software presented a single digit (an integer from 0 to 9) in white color on a black background at a random interval between 2 and 10 sec. Subjects were instructed to click the mouse button only if the number was odd and not click if the number was even. If the mouse was clicked on an odd number or if the mouse was not clicked on an even number for two sec, the number disappeared and the next number was displayed after a randomized interval. If the mouse was not clicked on an odd number within two sec or if the mouse was clicked on an even number, the number was displayed in red until the mouse was clicked (when the number was odd) or 2 sec passed (when the number was even). Then, the next number was displayed after a randomized interval. The test continued for 15 min, and the average RT and frequency of minor lapse (>500 ms) during the first, second, and last 5 min were calculated separately. To evaluate the sustainability of concentration, the ratios of the average RT and lapse frequencies of the second and last 5 min against those of the first 5 min were calculated as %RT 2 and 3, and %Lapse frequency 2 and 3, respectively.

### Subjective comfort

We used a visual analog scale (VAS) to compare subjective comfort between the two different combined lightings. We developed a VAS scale with 13 pairs of adjective phrases related to impression and mood at both ends of the linear scale; the adjective phrase pairs included dark and bright, hot and cool, powerless and powerful, feel bad and feel good, depressed and refreshing, not motivating and motivating, uncomfortable and comfortable, sleepy and awake, feel passive and feel active, restless and calm down, attention decreases and attention increases, tired and fine, and hard to see and easy to see. At the end of each combined light exposure, subjects were asked to mark positions on the VAS scales from 0 to 5, depending on their subjective feelings.

### Statistical analysis

Statistical analyses system version 9.4 (SAS institute Inc., Cary, NC, USA) was used for the statistical analysis. The effects of the type of combined lighting on the indices of autonomic functions, psychomotor performances, and subjective comfort were evaluated by the mixed-model analyses of variance using SAS Mixed procedure. Lighting type, age (young or old), exposure order, and interaction between lighting type and age were included in the model as the fixed effects and subject as random effect. In the figures and tables, the results of the analysis were presented as least-square means adjusted for the effect of order, unless otherwise noted. *P* <0.05 was considered to be statistically significant.

## Results

**Fig. 3:**
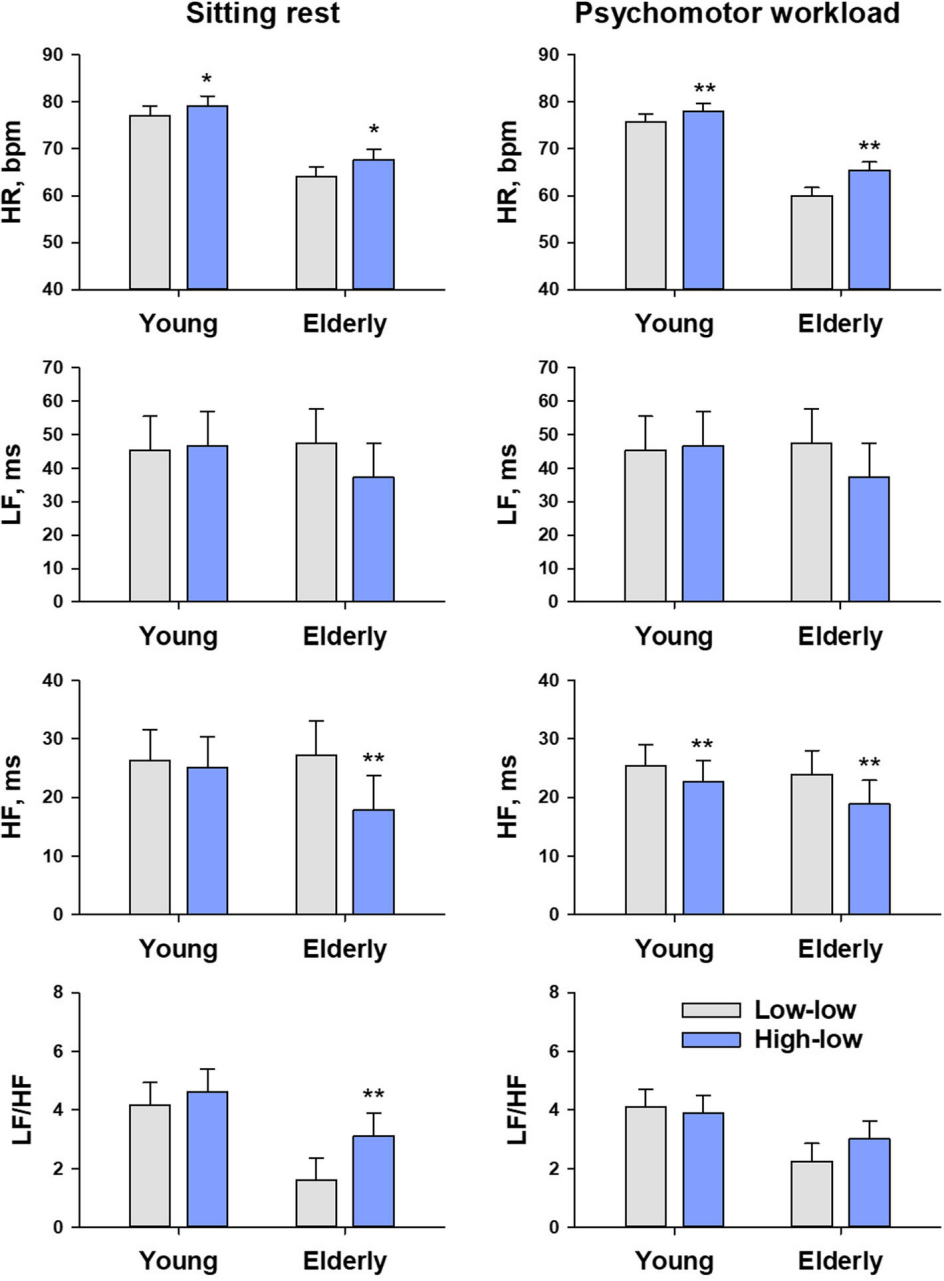
Autonomic function indices of heart rate variability at sitting rest and during psychomotor workload under the two types of combined lighting. Data are least-square means and the standard errors of the means (error bars) adjusted for the effects of experimental order. Low-low = low-CCT (4500 K) ambient lighting+ low-CCT (5000 K) task lighting; high-low = blue-enriched high-CCT (12000 K) ambient lighting + low-CCT (5000 K) task lighting. *P <0.05 and **P <0.01: The significance of difference between the low-low and high-low combined lightings within each age group

Figure 3 shows the least-square mean of autonomic function index of heart rate variability at sitting rest and during psychomotor workload under the two types of combined lighting. At sitting rest, significant main effects of lighting type were observed on heart rate (*P* = 0.01), HF amplitude (*P* = 0.0001), and LF/HF (*P* = 0.01), although there was a significant interaction between lighting type and age on HF amplitude (*P* = 0.0002). Post-hoc multiple comparisons revealed that heart rate increased in both young and elderly groups and HF amplitude decreased only in elderly group under the high-low combined lighting than the low-low combined lighting. During psychomotor workload, significant main effects of lighting type were observed on heart rate (*P* <0.0001) and HF amplitude (*P* <0.0001), although there was a significant interaction between lighting type and age on heart rate (*P* <0.0001) and HF amplitude (*P* = 0.01). Post-hoc multiple comparisons revealed that heart rate increased in both young and elderly groups and HF amplitude decreased only in elderly group under the high-low combined lighting than the low-low combined lighting.

**Fig. 4:**
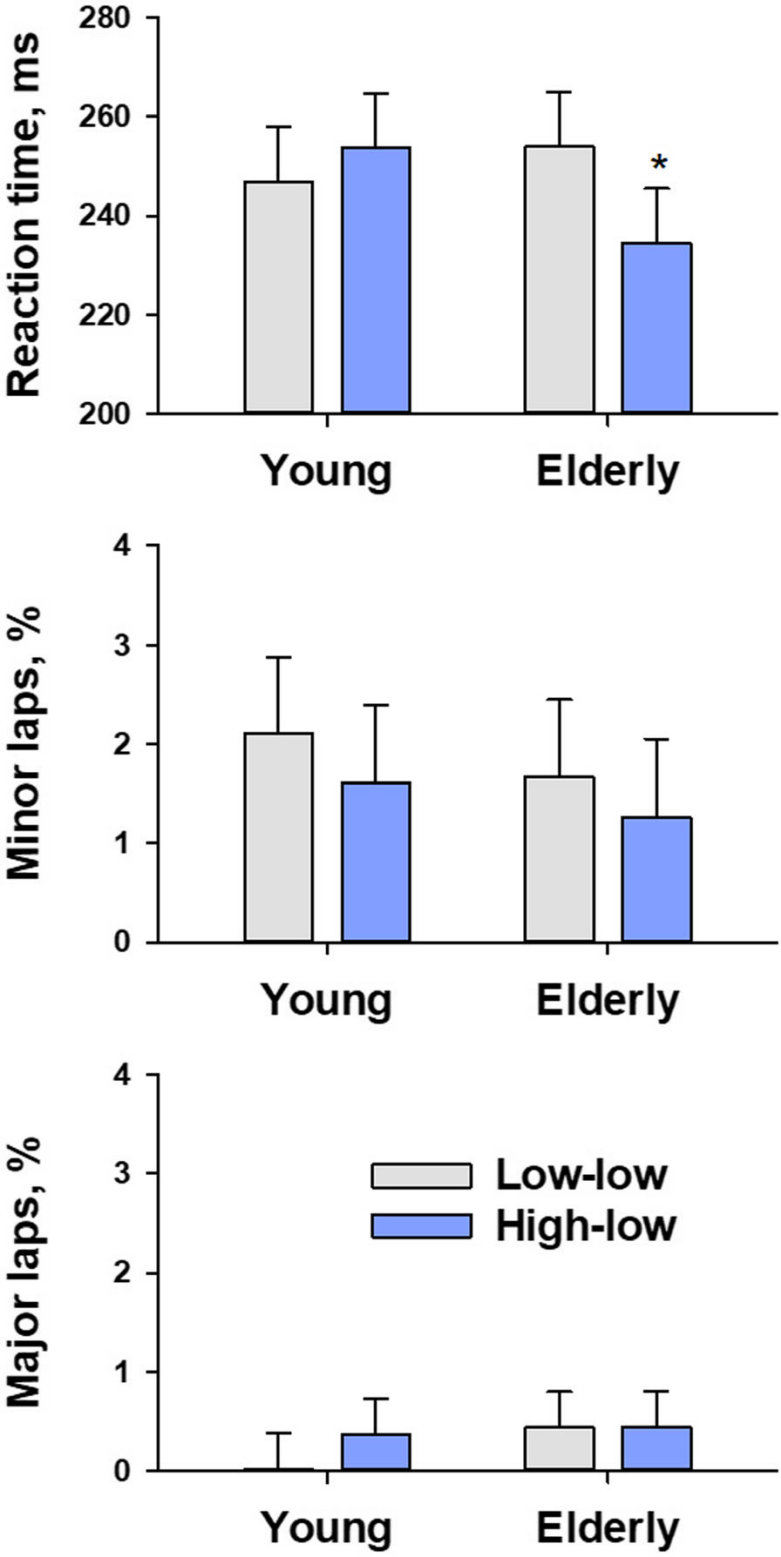
PVT performance indices under the two types of combined lighting. Data are least-square means and the standard errors of the means (error bars) adjusted for the effects of experimental order. * *P* <0.05: The significance of difference between the low-low and high-low combined lightings within each age group

Figure 4 shows the PVT performance indices under the two types of combined lighting. Although no significant main effect was observed for the lighting type on any PVT indices, a significant interaction between lighting type and age was observed for RT (*P* = 0.04). Multiple comparisons revealed that the high-low combined lighting shortened the RT only in the elder group but had no significant effect in the young group.

Table 3 shows the dependence of PS-PVT and VCPT performance on the types of combined lighting. Although age group showed main effects on RT (*P* = 0.005) and lapse frequency (*P* = 0.003), the type of combined lighting showed no significant main effect on the PS-PVT performance. Significant main effects of age group were also observed for the RTs of VCPT (*P* = 0.02, 0.02, and 0.03 for RT1, RT2, and RT3, respectively). Although no significant main effect on lapse frequency was observed for either combined lighting type or age, a significant main effect of lighting type was observed on the %lapse frequency of the last 5 min (*P* = 0.04). The lapse frequency was decreased in the last 5 min under the high-low combined lighting, while it was unchanged or increased under the low-low combined lighting.

**Table 3:** Dependence of psychomotor performances on the types of combined lighting

	Young		Elderly	
	Low-low	High-low	Low-low	High-low
*Pedal-selective PVT (PS-PVT)*				
RT, ms	411 ± 49	415 ± 49	654 ± 49	695 ± 49
False pedaling, %	6.8 ± 2.2	11.7 ± 2.2	5.5 ± 2.2	4.4 ± 2.2
Lapse frequency, %	3.6 ± 2.9	2.6 ± 2.9	14.3 ± 2.9	16.5 ± 2.9
Correction time, s	1.2 ± 1.4	0.8 ± 1.4	5.2 ± 1.4	2.0 ± 1.4
*Visual continuous performance test (VCPT)*				
RT 1, ms	516 ± 23	515 ± 23	609 ± 23	602 ± 23
RT 2, ms	511 ± 26	510 ± 26	616 ± 26	605 ± 26
RT 3, ms	538 ± 26	518 ± 26	611 ± 26	613 ± 26
%RT 2, %	99 ± 2	99 ± 2	101 ± 2	101 ± 2
%RT 3, %	104 ± 3	101 ± 3	100 ± 3	102 ± 3
Lapse frequency 1, %	3.3 ± 0.7	4.2 ± 0.7	2.7 ± 0.7	3.6 ± 0.7
Lapse frequency 2, %	3.4 ± 1.3	3.6 ± 1.3	2.6 ± 1.3	1.4 ± 1.3
Lapse frequency 3, %	2.7 ± 1.3	2.4 ± 1.3	4.1 ± 1.3	1.3 ± 1.3
%Lapse frequency 2, %	123 ± 28	68 ± 28	96 ± 28	36 ± 28
%Lapse frequency 3, %	96 ± 29	65 ± 29	141 ± 29	29 ± 29

Data are least-square means ± standard errors of the means adjusted for the effect of experimental order. Low-low = low-CCT (4500 K) ambient lighting+ low-CCT (5000 K) task lighting; High-low = blue-enriched high-CCT (12000 K) ambient lighting + low-CCT (5000 K) task lighting; RT = reaction time; %RT i = 100 x (RT i)/(RT 1); %Lapse frequency i = 100 x (Lapse frequency i)/(Lapse frequency 1). RT = reaction time.

**Fig. 5:**
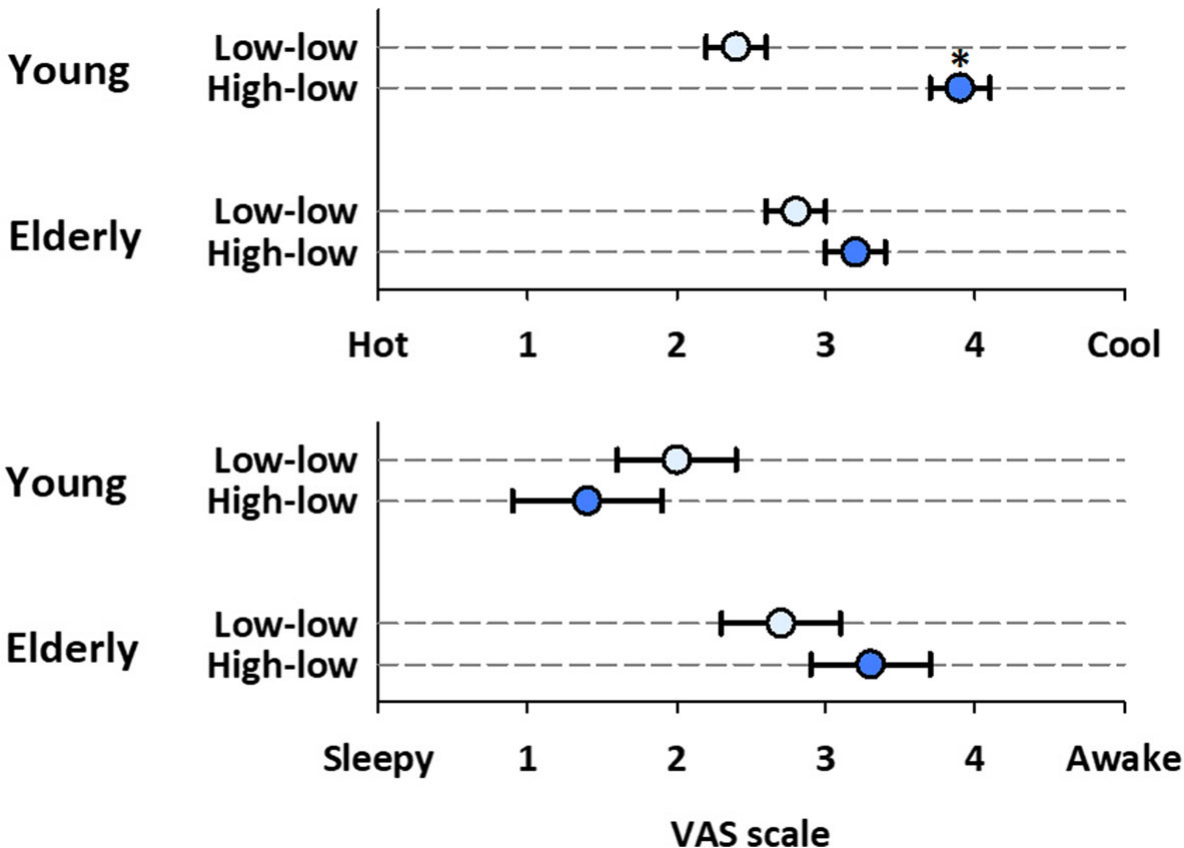
Visual analog scale scores of subjective feelings under the two types of combined lighting. Only scale scores of adjective phrase pair with significant main effects of lighting type (hot and cool) and of age (sleepy and awake) are shown. Data are least-square means and the standard error of the means (error bars) adjusted for the effects of experimental order. * *P* <0.05: The significance of difference between the low-low and high-low combined lightings within each age group

Figure 5 shows the VAS scores of subjective feelings that showed significant main effects of lighting type or age. A significant effect of lighting type was observed on the adjective phrase pair of hot and cool alone (*P* = 0.002) and there was a significant interaction between lighting type and age on the pair (*P* = 0.02). Post-hoc multiple comparisons revealed that the young subjects felt cooler under high-low combined lighting than low-low combined lighting, but elderly subjects felt no significant difference. Additionally, a significant main effect of age was observed on the adjective phrase pair of sleepy and awake (*P* = 0.01). The results of multiple comparisons showed that regardless of the type of lighting, young subjects felt more sleepy than elderly subjects.

Other than these, no significant difference in subjective feeling was observed between the two types of lightings and there was no significant interaction between age and lighting type in any of the subjective feeling scores. Additionally, none of the subjects reported preferences or complaints about the differences between the two types of lightings, and no subjects explicitly reported subjective color differences between them.

## Discussion

In this study, we investigated whether adjusting the CCT of ambient light alone in combined ambient-task lighting can achieve biologically favorable lighting without compromising subjective comfort in healthy subjects. We observed that high-CCT ambient lighting, even when used in combination with low-CCT task lighting, increased autonomic arousal level in both young and elderly subject groups, increased psychomotor vigilance in the elderly, and improved sustainability of concentration task performance in both age groups. The effect of high-CCT ambient lighting on autonomic arousal was more pronounced in elderly subjects. The difference in CCT in ambient lighting did not have a substantial effect on subjective feeling; particularly in elderly group, there was no significant difference in the VAS scores of any subjective feelings between the different CCTs of ambient lighting. Our findings suggest the possibility of adjusting the CCT of ambient light alone in combined ambient-task lighting as a way to achieve both bioregulatory function and subjective comfort.

There are many earlier studies reporting biological effects of high-CCT lighting [2–4, 7, 9, 26, 27] and blue light [17–20, 25]. Deguchi et al. [2] reported increased mental activity and Kobayashi et al. [3] reported increased diastolic blood pressure with 7500 K lighting compared with 3000 and 5000 K lighting. Yasukouchi et al. [7] examined the effect of CCT of lighting on body temperature in a moderately cold environment and observed a greater reduction in skin temperature under 3000 K lighting than under 5000 K and 7000 K lighting. Kozaki et al. [26] reported reduced stage-4 sleep amount during the early sleep phase and Ishibashi et al. [27] reported decreased HF component of heart rate variability during sleep with exposure to 6700 K light 6.5 h before sleep compared with 3000 K light. As to the effects of blue light, Higuchi et al. [18] reported that the cutting of short-wavelength light by a red-visor cap prevented melatonin suppression by nighttime (23:00-03:00) light exposure that was observed under the use of a non-visor cap. Yuda et al. [19, 20] reported suppression of HF component during exposure and enhanced PVT performance after the exposure to blue monochromatic light compared with red, green, and even white lights. Consequently, many authors have also alerted the sleep and circadian rhythm disturbance by the nighttime use of smartphones and tablet devices that emit blue light [28–30, 39–41].

Our present observations were in the same line as these earlier reports, but we demonstrated that the effects of blue-enriched high-CCT light could also appear when it is used as ambient lighting together with low-CCT task lighting. Our observations suggest that high-low composite lighting has autonomic and psychomotor arousal effects. Compared with the low-low combined lighting, heart rate was higher and HF amplitude was lower both at sitting rest and during psychomotor workload under the high-low combined lighting. These changes are consistent with a decrease in cardiac vagal activity associated with increased autonomic arousal levels [42]. Also, in the elderly group, the RT of PVT shortened under the high-low combination lighting. This indicates increased sustained attention in the elderly due to increased levels of psychomotor arousal [43]. Additionally, in both age groups, the lapse frequency of the last 5 min of VCPT reduced under the high-low combined lighting, while it was unchanged or increased under the low-low combined lighting. This suggests improvement of the sustainability of concentration works under the high-low combined lighting.

These arousal effects of high-low combined lighting were more pronounced in elderly than in young subjects. Although the exact mechanisms cannot be determined from this study, at least two possibilities may be speculated. First, the autonomic and psychomotor arousal effects of blue light through the ipRGC system may increase with age. The activity of ipRGC system can be assessed by means of pupil responses to bright blue (around 480 nm) light [44, 45]. Herbst et al. [46] found that the pupil responses to blue light via ipRGC increases with age, even though the amount of blue light that passes through the lens of the eye and illuminates the retina decreases with age. Other studies, however, reported the stability of the pupil response across human lifespan [47, 48]. Second, age-related changes in lens properties may increase the distribution of blue light in the retina. With aging, lens proteins undergo both physiological and pathological modifications that increase light scattering in the eyes [49], which might increase the retinal area illuminated by blue light. Whatever the mechanisms, however, the findings of the present study suggest that the use of the combined lighting may help maintain daytime alertness and psychomotor performance in older workers and older people who are difficult to expose to outside light.

Finally, we observed that the CCT of the ambient light of the combined lighting did not affect subjective comfort substantially. The VAS assessment of subjective feelings indicated that the impression of the lighting and mood felt under lighting did not differ between the high-low combined and low-low combined lightings, except that the young subjects felt cooler under the high-low combined lighting. None of the subjects reported preferences or complaints about the differences between them, including color. In this study, we devised the ambient lighting panel that illuminates from a direction perpendicular to the subject's visual axis so that the ambient light enters the subject's retina only from the upper visual field. Lasko et al. [44] reported that nighttime bright white light in the upper visual field caused greater melatonin suppression than the light in the lower visual field. Glickman et al. [45] also demonstrated that nighttime white light exposure to the inferior retina caused melatonin suppression, while exposure to the superior retina caused no significant suppression. In a previous study, we also investigated the effect of the angle of incidence on the autonomic effects of blue light and observed similar effects with light from directly above [31]. The present observations indicate that the biological effects of the non-imaging function of blue light could be regulated separately from the subjective comfort caused by the image-forming function by choosing different directions of light into the eye for ambient and task light.

There has been increasing interest in the use of lighting to maintain and promote health and well-being in-home, facility, and office environments [25, 50–52]. The lighting required from the aspects of health and well-being, however, often does not match subjectively comfortable lighting or lighting required for work and study. The present study shows the possibility of solving this problem by controlling the CCT of ambient light for bioregulation, while maintaining the task lighting necessary for individual subjective comfort and various activities.

**Limitations**

This study has several limitations. First, our results indicated that high-CCT ambient lighting induces increases in autonomic arousal, elderly psychomotor vigilance, and concentration sustainability, but it is unclear whether ambient lighting with a CCT below 4500 K, when used with 5000 K task lighting, can reverse these effects. Second, we examined the effects of the combined lighting only on the autonomic and psychomotor arousal levels and subjective comfort, but did not examine other non-image forming effects such as sleep quality and circadian rhythms. Third, there are circadian rhythms in the arousal level of autonomic and psychomotor functions. To take into account the effect of clock time, all experiments were conducted between 13:00 and 16:15, but the effect of light on the arousal level may vary depending on the time of day. Fourth, although no subjects explicitly reported subjective color differences between the two types of ambient light, it is possible that not only differences in CCT but also differences in color may be involved in the different effects on autonomic and psychomotor functions. In a previous study comparing autonomic function under red, green, and blue monochromatic light, we observed that only blue light increased autonomic arousal levels, with no difference between red and green light [20]. Fifth, the total illuminance of the combined lights was adjusted to 750 lx for all experimental conditions, but the results may vary depending on illuminance. Sixth, the high-CCT ambient light when used in combination with the low-CCT task light showed no substantial impact on subjective comfort, but, due to the output limitation of the luminance, we were unable to compare it directly to the subjective comfort of lighting with the same illuminance with only a single high-CCT light. Seventh, the height of the ambient light was lower than that of usual ceiling lights and the angle of incidence on the eyes may have been sensitive to changes in the position of the subject's head. As shown in Figure 1, however, we devised the arrangement of ambient lighting so that it would illuminate the subject’s eyes from directly above or behind when the subject looks at the computer display. Finally, we observed that the older subjects had greater autonomic and psychomotor arousal effects of the high-low combined lighting than young subjects, but the number of subjects was small and the groups were sexually imbalanced. Also, since the present observations were obtained with a single type of LED lighting. Future studies need to investigate the generalizability.

## Conclusions

High-CCT ambient light entering the eye from a direction perpendicular to the visual axis, even when used in combination with low-CCT task lighting, could increase autonomic and psychomotor arousal levels without compromising subjective comfort. This finding suggests the feasibility of independent control of ambient and task lighting as a way to achieve both biological function regulation and subjective comfort.

## Abbreviations

ECG: electrocardiogram
CCT: correlated color temperature
HF: high frequency
ipRCG: intrinsically photosensitive retinal ganglion cells
LED: light-emitting diode
LF: low frequency
LF/HF: LF-to-HF ratio
PS-PVT: pedal-selective psychomotor vigilance test
PVT: psychomotor vigilance test
RT: reaction time
VAS: visual analog scale
VCPT: visual continuous performance test
